# Frequency-dependent nanomechanical profiling for medical diagnosis

**DOI:** 10.3762/bjnano.13.122

**Published:** 2022-12-09

**Authors:** Santiago D Solares, Alexander X Cartagena-Rivera

**Affiliations:** 1 Department of Mechanical and Aerospace Engineering, The George Washington University, School of Engineering and Applied Science, Washington, District of Columbia, USA; 2 Section on Mechanobiology, National Institute of Biomedical Imaging and Bioengineering, National Institutes of Health, Bethesda, Maryland, USA

**Keywords:** atomic force microscopy, healthcare, mechanical properties, mechanobiology, medical diagnosis

## Abstract

Atomic force microscopy (AFM), developed in the early 1980s, has become a powerful characterization tool in micro- and nanoscale science. In the early 1990s, its relevance within biology and medicine research became evident, although its incorporation into healthcare applications remains relatively limited. Here, we briefly explore the reasons for this low level of technological adoption. We also propose a path forward for the incorporation of frequency-dependent nanomechanical measurements into integrated healthcare strategies that link routine AFM measurements with computer analysis, real-time communication with healthcare providers, and medical databases. This approach would be appropriate for diseases such as cancer, lupus, arteriosclerosis and arthritis, among others, which bring about significant mechanical changes in the affected tissues.

## Introduction

Since its invention in the early 1980s, atomic force microscopy (AFM) has been extensively used for topographical, mechanical, electrical, and chemical characterization of surfaces ranging from semiconductors and metals to polymers and biological materials [1–5]. In particular, a variety of mechanical property measurement methods have been developed, although most of them are restricted to relatively simple physical descriptions, such as an elastic modulus or qualitative measures [6–13], which cannot always be unambiguously interpreted for biological specimens. There also exist a few methods based on linear viscoelasticity, which can be used to estimate frequency-dependent quantities, such as the storage and loss modulus [14–17]. These quantities are appropriate for characterizing soft viscoelastic materials, such as biological specimens, whose mechanical response depends on the rate of application of stress or strain. Notably, many measurements on complex biological systems are still reported using quantities based on elastic approximations, which are not capable of properly reproducing their mechanical behavior [16,18–19].

Despite significant progress in the development of sophisticated AFM methodologies, broad adoption of these methods in high-impact biological and healthcare applications remains relatively limited. In this perspective article, we discuss some of the reasons for this limited adoption and build on previous measurements performed on healthy and diseased cells and tissues to propose a possible path for the incorporation of AFM nanomechanical measurements into healthcare strategies related to diseases or conditions that are associated with mechanical changes in the tissues involved. Some examples include cancer, arteriosclerosis, lupus, arthritis and glaucoma, among others. Within this strategy we highlight the development of application-specific sensors, real-time communication with healthcare providers, as well as the development of large databases of robust measurements classified by patient biometrics or as a function of disease progress.

## Perspective

### Limited adoption of nanomechanical AFM measurements in broad-impact medical applications

AFM is a well-established and widely used technique for fundamental micro- and nanoscale research, especially concerning topographical characterization and general force measurements [1–5]. However, advanced mechanical property analysis is not yet widely used for broad-impact applications. In fact, while some mechanical property AFM studies have enjoyed relatively good readership, most of their citations come from fundamental research papers, suggesting that their level of technological adoption in industrial and healthcare applications may still be relatively low. This is also true for clinical settings, where the bench-to-bed connection has not yet been clearly established, despite a variety of mechanobiological studies for diseases such as cancer [18,20] (we do recognize that industrial adoption of AFM methods does not necessarily result in citation of academic papers and are aware that AFM methods have been used in corporate or corporate-funded research for the characterization of polymers, semiconductors and other materials [21–29]. It is also well known that we owe the invention of the AFM to scientists at IBM [1]).

**Figure 1:**
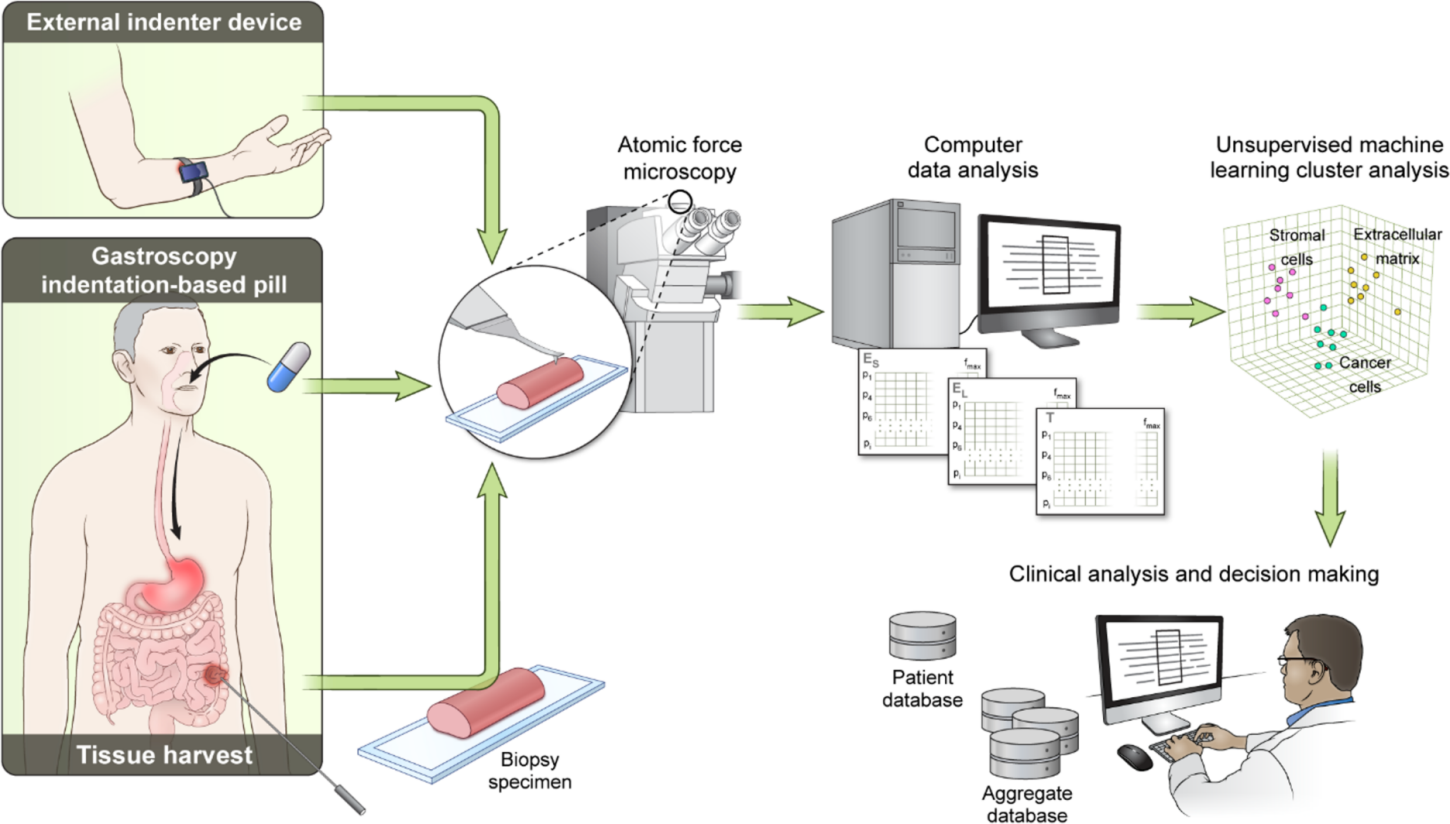
Example of nanomechanical profiling strategy of patient tissues for medical diagnosis. Multiple non-invasive and invasive indentation-based strategies are proposed to characterize the frequency-dependent material properties of multiple patient tissues depending on accessibility and disease. Note that the proposed indentation strategies are not mutually exclusive. For example, intestinal tissue harvesting for characterization on an external AFM device may follow an initial scan conducted with an indentation-based pill (Figure 2) if the latter method raises serious concerns about the patient’s health. After acquisition of tissue physical data, a computer data analysis is performed to determine the frequency-dependent mechanical properties (e.g., *E*_S_ and *E*_L_). Then, an unsupervised machine learning cluster analysis is performed to identify important features (“biomarkers”) within the data. Finally, the clinician correlates the obtained patient mechanical data with the patient demographics and an aggregate database to make a decision concerning disease stage and therapeutic approaches. Nomenclature: *E*_S_ – storage modulus, *E*_L_ – loss modulus, T – topography, *f* – frequency, and p – points within nanomechanical maps.

The slow adoption of AFM methods in broad-impact applications can be explained by a number of reasons. First, ambiguity is generally present in the assumptions, methods, and data analysis of AFM measurements, which are limited to the interaction of a probe of relatively simple apex geometry (e.g., a spherical tip) with a surface, whose properties may differ from those of the material below it. Second, the results are not always transferrable, since they are often not expressed in rigorous physical units. Specifically, for mechanobiological AFM measurements, it is quite common to only estimate the Young’s modulus of the material. However, this quantity is not defined for viscoelastic materials (biological tissues exhibit viscoelastic behavior), whose mechanical response depends on the rate of deformation. Third, AFM mechanical characterization is not always fully repeatable and can depend on equipment, sample preparation, and user expertise (or “art”). Further, there is often no direct connection to specific technological applications. For example, multiple studies have shown that the mechanical properties of cancerous cells differ from those of healthy cells [15,20], but it is not clear how those measurements could be exploited for medical treatment. This is in sharp contrast to typical macroscale applications where, for example, the mechanical properties of steel and concrete can be directly used by engineers to design a bridge and predict its performance under real-life conditions. Finally, there is not sufficient communication between AFM experts (typically physicists or engineers) and application “customers”. For instance, in the context of cancer, there is still relatively limited collaboration among engineers, biophysicists, cancer biologists, and oncologists, which impedes progress in elucidating the role of mechanics in disease progression, and within monitoring and treatments such as chemotherapies and immunotherapies. In our opinion, closer collaboration of the above disciplines would enable rigorous nanomechanical studies of cancer cells and tumor microenvironments in controlled physiologically relevant conditions. This could also enable performance analyses that could be extended to more complex and relevant scenarios, aided by advanced analysis tools such as machine learning (see below in Figure 1).

### Decoding frequency-dependent nanomechanical measurements for disease study and follow-up

The broad adoption of AFM nanomechanical measurements would require their integration into streamlined strategies for addressing meaningful and far-reaching applications, such that the measurement provides information that is directly applicable, beyond long-term scientific relevance. As has often been proposed, we believe that this may be most easily achieved in the healthcare field, where AFM can be integrated into in vivo measurements through devices that fit the particular application (as opposed to a generic commercial AFM) and are capable of (i) performing repeated routine characterization directly on patient tissues and (ii) providing immediate results that can be used to unambiguously evaluate disease progress. It is also necessary that the measurement device only require minimal training on the part of the user, which may be the physician or even the patient (at home). For non-topical tissue measurements, it would be necessary to establish a strict standard procedure for biopsy analysis or for the device to reach the affected tissue inside the body (for example, by means of a catheter inserted into a vein, or through the collection of a biopsy from arthritic tissue). In all cases, such an integrated approach would enable the generation of a database with rigorous nanomechanical measurements in a variety of healthy and diseased tissues, either classified by demographics and biometric factors, or according to disease progress for a specific patient. Figure 1 exemplifies an overall strategy for integrating AFM-based nanomechanical measurements into medical diagnosis, which includes specialized in vivo sensing, computer analysis (AFM force spectroscopy analysis), communication with the healthcare provider, and mining of the aggregate community database.

**Figure 2:**
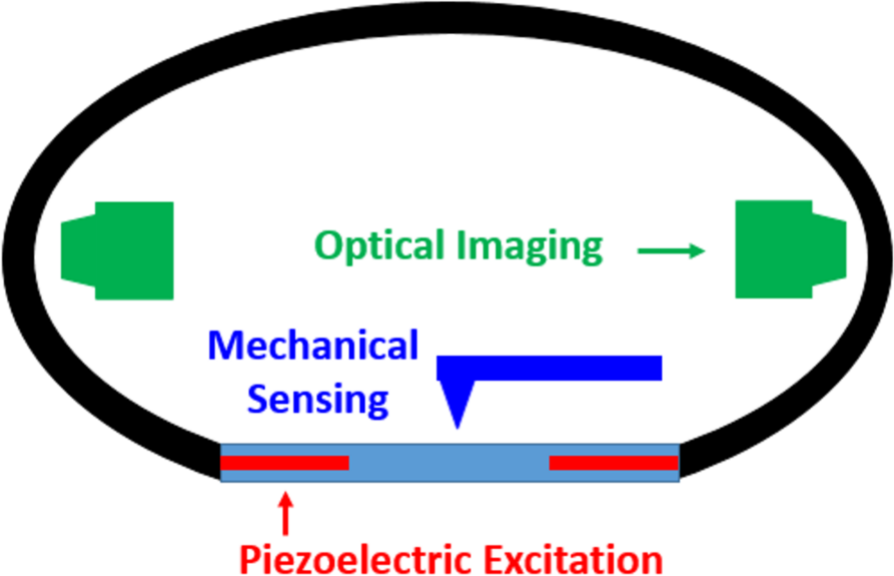
Schematic of proposed enhanced endoscopy pill. The design is based on existing devices [30] that perform optical imaging, whose capabilities could be augmented to perform mechanical sensing, for example, through the incorporation of a piezoelectrically actuated membrane equipped with a mechanical response sensing mechanism [31–32].

Given the mechanical nature of AFM, the above strategy naturally fits diseases that are linked to mechanical changes in cells and/or the extracellular matrix. Existing micro-robotics technology could be harnessed to develop the specific types of sensors needed in each case. For example, in the case of gastrointestinal cancer screening, one could envision the integration of a mechanical sensor into an endoscopy pill, which would save or transmit its mechanical data to a computer for further analysis. Endoscopy capsules for optical imaging of the digestive tract already exist [30]. A schematic for a proposed enhancement to this type of device is shown in Figure 2. In addition to optical imaging, the device could be equipped with one or more piezoelectrically excited membranes coupled with a sensing mechanism, such as an AFM cantilever or other type of mechanical sensor (similar stand-alone developments already exist [31–32]). The mechanical response of the membrane could be measured while it is in close contact with the surrounding tissue. Similar developments could be envisioned for measurements inside veins or arteries, in which case the sensing device could be inserted by means of a catheter, such as those already in use [33]. In this case, greater miniaturization would be required, but a suitable design seems plausible within the next decade. Finally, for external measurements, such as in the case of skin cancer measurements, a hand-held, non-invasive indenter could be brought up directly to the lesion. The latter example requires the least technological development, as there is no need for the device to be as small as in the previous two examples. Intermediate situations include, for example, characterization of arthritic tissues, for which biopsies can be obtained relatively easily and analyzed externally or for which the affected tissues can be reached inside the body through small insertions. It is also worth mentioning that the AFM measurement principle does not need to be limited to the use of microscale or nanoscale probes. In fact, the mathematics of the viscoelastic contact problem between a probe of known geometry (e.g., a sphere) and a flat surface [15–17] could be applied at different length scales, ranging from the nano- to the macroscale. One can envision various applications at larger scales, such as the characterization of muscular viscoelasticity in orthopedic rehabilitation or in athletics, where the mechanical properties of muscles, tendons, ligaments, and bones have a direct impact on the patient’s locomotion or athletic performance. As the length scale of the probe increases, so do the probe–sample interaction forces observed during the measurement, and the characterization returns greater mechanical information about deeper and deeper layers of the sample. In contrast, as the length scale of the probe decreases, the mechanical information obtained is restricted to thinner and thinner regions near the sample surface.

**Figure 3:**
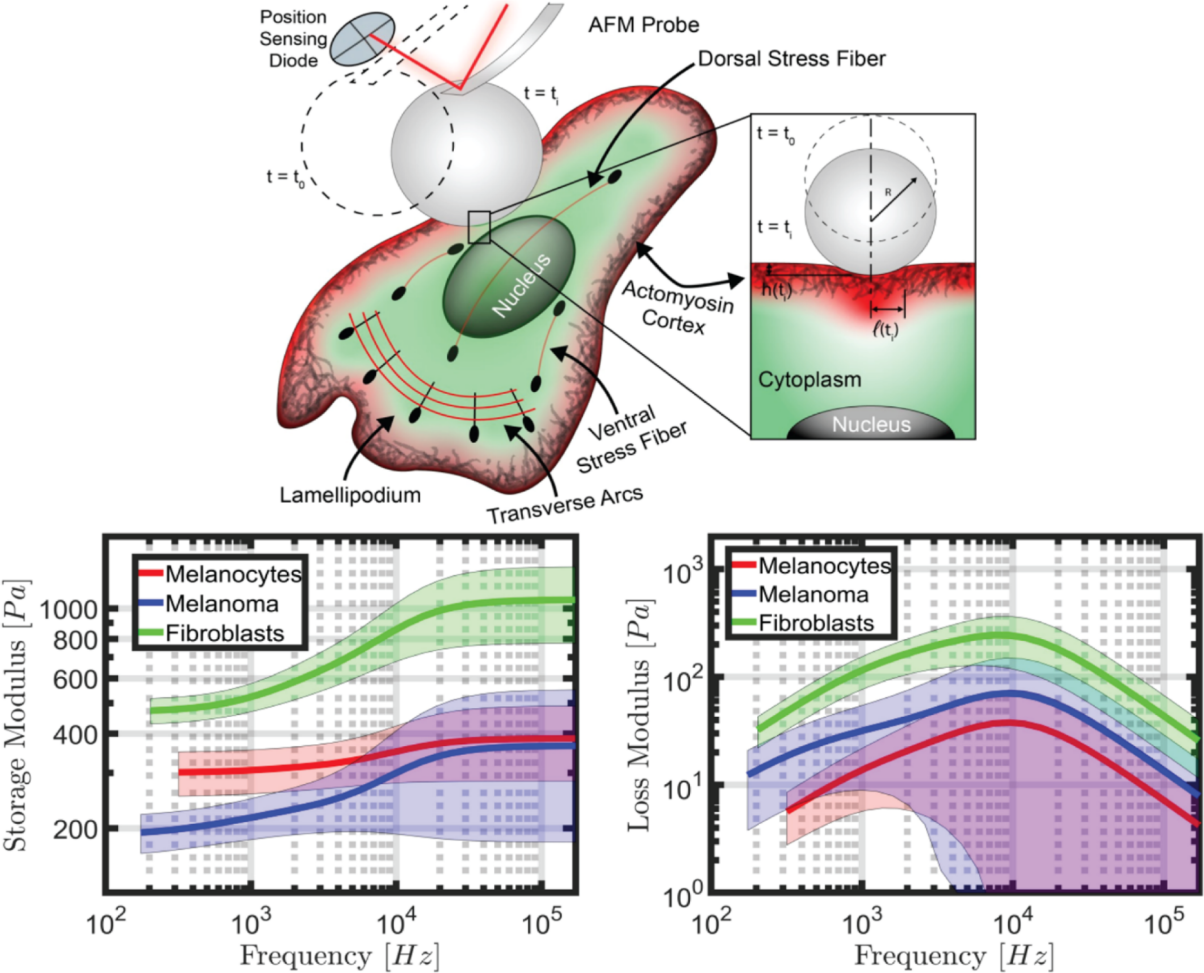
Illustration of a 2D adherent cell indented by a micrometer-sized spherical AFM probe, as well as storage and loss modulus calculated from the parameterized Generalized Maxwell model for 2D adherent normal and cancerous human skin cells, fitted from AFM force–distance curves (Adapted from [15] © 2022 C. H. Parvini et al., published by Springer Nature, distributed under the terms of the Creative Commons Attribution 4.0 International License, https://creativecommons.org/licenses/by/4.0)).

In order to address the measurement ambiguity described in the previous section, we propose that the result of the AFM analysis for a viscoelastic material needs to be expressed in the appropriate physical quantities, namely the retardance or relaxance of the material or equivalent frequency-dependent quantities [15–17]. These are rich, transferrable quantities that offer much more detailed information than a single scalar quantity such as a modulus of elasticity, and from which traditional viscoelastic quantities can be obtained, such as the storage and loss modulus (which are also frequency dependent). Figure 3 provides an example of storage and loss modulus estimates for cancerous human melanoma cells as well as healthy human melanocytes and fibroblasts, showing that clear cell differentiation is possible when a wide frequency range is considered instead of a single number such as a pseudo-Young’s modulus. Furthermore, such representations would be extremely useful to evaluate the gradual evolution of cell mechanical properties as a particular disease progresses, as the patient ages, or as a function of other biometric characteristics. For example, as tissues age or become diseased, one may observe changes in the complex modulus in some specific regions of the frequency axis, but not in others. Small changes in the shape of the curve could signal or confirm the onset of disease, perhaps even before clinically detectable symptoms emerge. This type of evaluation is difficult (or nearly impossible) to perform when a pseudo-Young’s modulus is used, since the latter is a single quantity that neglects the viscoelastic nature of the material and which can depend on equipment and measurement parameters. Interestingly, it is still common in medical research in general (not just in the context of nanoscale measurements) to neglect the viscoelastic properties of tissues and to focus only on elastic property approximations, for example in the study of arterial degeneration [34–35].

Besides the above frequency-dependent representation, other important measurements can be recorded, such as general topography and morphology, cell or tissue adhesive properties, internal hydrostatic pressure, and surface tension. These additional multidimensional biophysical parameters could be inferred from the AFM spectroscopy data as well, and could be correlated with the frequency-dependent nanomechanical parameters previously described to provide richer mechanistic knowledge of the genesis of a complex disease and its progression.

### A possible path for the feasible propagation of viscoelastic AFM measurements into medical practice

While the connection between nanomechanical AFM measurements and clinical applications seems intuitive, the transfer of technology into clinics is not straightforward and requires a well-coordinated effort. To this end, we propose the formation of multidisciplinary project teams, as described below, each of which would focus on the evaluation of AFM methods in the context of a specific disease. Naturally, it would be beneficial to have multiple such teams, as the use of AFM may or may not be something that needs to be implemented at the clinic or hospital level in all cases evaluated.

First, we propose that the team be composed of at least the following members in order to have expertise in all the relevant areas of technology transfer: an AFM scientist who is committed to the implementation of rigorous viscoelastic analysis methods that correspond to the true physical behavior of biological systems (i.e., not elastic or pseudo-elastic treatments), a scientific instrumentation expert who can build and/or modify the required devices, a medical researcher, a clinician, a computer scientist, and an industrial partner. Second, we propose that each team focuses on only one disease at a time, in order to explore deeply whether the proposed technology is truly beneficial in the evaluation and treatment of that particular disease (the objective of the project should not be to force the technology into the clinical application, but rather to evaluate honestly and realistically whether there truly is a benefit that should be exploited). Third, following selection of the disease to be evaluated, we suggest that the project include a well-thought-out device development and measurement strategy, where the right type of instrument is designed and developed, which may or may not look like the typical AFM instrument, but which can acquire the desired type of nanomechanical information in the same type of transferrable physical units. Fourth, the team should develop a rigorous and honest plan to evaluate when and where there is value added when introducing the desired method, and such evaluation should be based on quantifiable indicators. Fifth, if a clear benefit is identified, the team could proceed to a pilot evaluation stage at the clinical or hospital level, where a database of patient information can begin to be developed and where the benefits of the previous evaluation can be confirmed or disproved. Finally, if the outcome still suggests that the transfer of technology should proceed, the team could then propose a strategy for further dissemination of the technology in medical environments and for its commercialization (Figure 1).

We realize that an integrated strategy such as the above would be very costly to implement and would also require time to execute. Furthermore, it would still need to be followed by a broad dissemination and commercialization effort and would require careful coordination to ensure that the various teams synergistically communicate with one another. Nevertheless, we believe that such efforts are worthwhile for improving medical diagnosis and follow-up, and for augmenting overall scientific medical knowledge, and encourage researchers and funding agencies to develop and pursue similar initiatives.

## Conclusion and Outlook

We have proposed a possible path for the incorporation of nanomechanical measurements performed with AFM into integrated healthcare strategies that link routine AFM measurements performed using application-specific sensors with computer analysis and real-time communication with healthcare providers. Such integration could be highly beneficial in the diagnosis and follow-up of diseases that cause observable mechanical property changes in the affected tissues, such as cancer, arteriosclerosis, lupus, arthritis and glaucoma, among others, as well as within rehabilitation or athletic settings where the mechanical properties of tissues directly influence the subject’s locomotion or athletic ability. Furthermore, such integration would also enable the creation of large standardized databases, from which important disease trends could be mined, which could in turn aid in the development of more advanced prediction and treatment methods than those currently available. Within the proposed strategy, recently developed frequency-dependent mechanical characterization methods play a central role, as they are the appropriate type of method for characterizing viscoelastic materials [15–17]. Although the required fundamental AFM developments are mostly available, along with the required computer analysis and communication infrastructure, it is important to point out that all previous frequency-dependent methods we are aware of are based on *linear* viscoelasticity. Since biological materials are known to be highly nonlinear, extension of existing methods into the nonlinear regime is highly encouraged. Additionally, application-specific AFM sensors still remain to be developed, although here also the required technologies are already available (e.g., microfabrication and micro-robotics).

While we have focused specifically on healthcare treatments of mechanically relevant diseases, similar technology adoption paths can be envisioned for other fields, such as tissue engineering, for which frequency-dependent characterization would be extremely beneficial. This would allow, for example, for the tailoring of artificial tissues to the type of stimuli associated with specific types of activities, such as job-related mechanical vibrations at different frequencies, sports-related accelerations, and other ergonomic factors. Similar considerations exist for the development of soft wearable sensors or hydrogels for wound healing or drug delivery, among others. In all cases, it is of prime importance to create project teams with the appropriate make-up of expertise and within a well-designed strategy, such that ideas can be seamlessly transferred from fundamental research to routine applications.
